# Impact of COVID-19 lockdowns on adolescent pregnancy and school dropout among secondary schoolgirls in Kenya

**DOI:** 10.1136/bmjgh-2021-007666

**Published:** 2022-01-13

**Authors:** Garazi Zulaika, Miriam Bulbarelli, Elizabeth Nyothach, Annemieke van Eijk, Linda Mason, Eunice Fwaya, David Obor, Daniel Kwaro, Duolao Wang, Supriya D Mehta, Penelope A Phillips-Howard

**Affiliations:** 1 Department of Clinical Sciences, Liverpool School of Tropical Medicine, Liverpool, UK; 2 UNU-MERIT/MGSOG, Maastricht University, Maastricht, The Netherlands; 3 Center for Global Health Research, Kenya Medical Research Institute, Kisumu, Kenya; 4 Siaya County, Kenya Ministry of Health, Siaya, Kenya; 5 Division of Epidemiology & Biostatistics, University of Illinois at Chicago, Chicago, Illinois, USA

**Keywords:** COVID-19, health policy, maternal health, public health

## Abstract

**Introduction:**

Secondary school closures aimed at limiting the number of infections and deaths due to COVID-19 may have amplified the negative sexual and reproductive health (SRH) and schooling outcomes of vulnerable adolescent girls. This study aimed to measure pandemic-related effects on adolescent pregnancy and school dropout among school-going girls in Kenya.

**Methods:**

We report longitudinal findings of 910 girls in their last 2 years of secondary school. The study took place in 12 secondary day schools in rural western Kenya between 2018 and 2021. Using a causal-comparative design, we compared SRH and schooling outcomes among 403 girls who graduated after completion of their final school examinations in November 2019 pre-pandemic with 507 girls who experienced disrupted schooling due to COVID-19 and sat examinations in March 2021. Unadjusted and adjusted generalised linear mixed models were used to investigate the effect of COVID-19-related school closures and restrictions on all outcomes of interest and on incident pregnancy.

**Results:**

At study initiation, the mean age of participants was 17.2 (IQR: 16.4–17.9) for girls in the pre-COVID-19 cohort and 17.5 (IQR: 16.5–18.4) for girls in the COVID-19 cohort. Girls experiencing COVID-19 containment measures had twice the risk of falling pregnant prior to completing secondary school after adjustment for age, household wealth and orphanhood status (adjusted risk ratio (aRR)=2.11; 95% CI:1.13 to 3.95, p=0.019); three times the risk of school dropout (aRR=3.03; 95% CI: 1.55 to 5.95, p=0.001) and 3.4 times the risk of school transfer prior to examinations (aRR=3.39; 95% CI: 1.70 to 6.77, p=0.001) relative to pre-COVID-19 learners. Girls in the COVID-19 cohort were more likely to be sexually active (aRR=1.28; 95% CI: 1.09 to 1.51, p=0.002) and less likely to report their first sex as desired (aRR=0.49; 95% CI: 0.37 to 0.65, p<0.001). These girls reported increased hours of non-school-related work (3.32 hours per day vs 2.63 hours per day in the pre-COVID-19 cohort, aRR=1.92; 95% CI: 1.92 to 2.99, p=0.004). In the COVID-19 cohort, 80.5% reported worsening household economic status and COVID-19-related stress was common.

**Conclusion:**

The COVID-19 pandemic deleteriously affected the SRH of girls and amplified school transfer and dropout. Appropriate programmes and interventions that help buffer the effects of population-level emergencies on school-going adolescents are warranted.

**Trial registration number:**

NCT03051789.

**Key questions**

**What is already known?**

- Experiences from past outbreaks show that emergency response policies have differential effects on girls and women, limiting their economic opportunities in the long term.
- Adolescent pregnancy constitutes a public health crisis in western Kenya, where over one in five girls enter motherhood during adolescence.

**What are the new findings?**

- In Kenya, adolescent secondary schoolgirls who remained out of school for 6 months due to the COVID-19 lockdown had twice the risk of becoming pregnant and three times the risk of dropping out of school when compared with similar girls graduating just prior to the outbreak.
- The sexual and reproductive health and schooling outcomes of these girls were affected in multiple ways, with girls reporting heightened sexual debut, sexual coercion and school transfer relative to girls who completed secondary school in the prior year.

**What do the new findings imply?**

- COVID-19 containment measures negatively affected vulnerable adolescent girls.
- Gender sensitive policy responses and interventions are needed to buffer the effects of health emergencies on individuals and communities.

## Introduction

The COVID-19 pandemic reached Kenya in March 2020, and with the first case came nationwide curfews, lockdowns and restrictions of movement. As part of the containment measures, the Kenyan Government shuttered all schools from March 2020 until January 2021 countrywide,^1^ disrupting education for millions of students. While all learners were affected, experts have voiced concern that the devastating social and economic costs will be felt most acutely by the most vulnerable students.^2 3^

In Kenya, media reports state that adolescent pregnancies have spiked due to COVID-19-related containment measures, raising concerns about the sexual and reproductive health (SRH) and longer-term schooling outcomes of this vulnerable population.^4 5^ Adolescent pregnancy already constitutes a major public health concern in Kenya; prior to COVID-19, one in five girls between 15 and 19 years was either pregnant or already a mother.^6^ Pregnancy early in a mother’s life course poses severe maternal and infant health risks.^7^ Social harms, such as child marriage, pregnancy-related stigma and community isolation, fear of school expulsion and lack of financial or emotional support, can lead pregnant adolescent girls to risk unsafe abortions, delay necessary healthcare or leave their communities altogether.^7 8^ These social harms can lead to poor mental health outcomes such as depression, anxiety and acute stress and the loss of income earning opportunities.^9 10^ Consequently, adolescent pregnancy is of major public health concern in Kenya and may serve as a proxy indicator reflecting girls’ vulnerability to population-level shocks such as COVID-19.

COVID-19-related school closures may have inadvertently magnified girls’ barriers to education and SRH vulnerabilities. Evidence suggests that staying in school de facto protects girls against early marriage, pregnancy and sexual and reproductive tract infections such as HIV.^11–13^ Moreover, once schools reopen, factors that reduce girls’ ability to remain in school stem from these same SRH harms (ie, pregnancy), poverty and gender inequality.^11 13 14^ In prior epidemics such as the west-African Ebola outbreak, staying home increased girls’ risk of domestic violence, intimate partner violence and abuse.^15 16^ Ebola-related school closures also decreased school attainment for female learners with girls attending 1.8 years of secondary prior to the epidemic compared with 0.9 years post epidemic.^17^ In this study, we examine whether COVID-19-related restrictions and school closures were associated with risk of school dropout, adolescent pregnancy and other SRH harms among secondary schoolgirls in rural western Kenya. It investigates data collected in 12 schools for the Cups or Cash for Girls (CCG) cluster randomised-controlled trial (cRCT) to compare these outcomes among girls who graduated in late 2019 with girls who would have graduated a year later but whose schooling was interrupted by the pandemic.

## Methods

### Study design and participant recruitment

**Figure 1:**
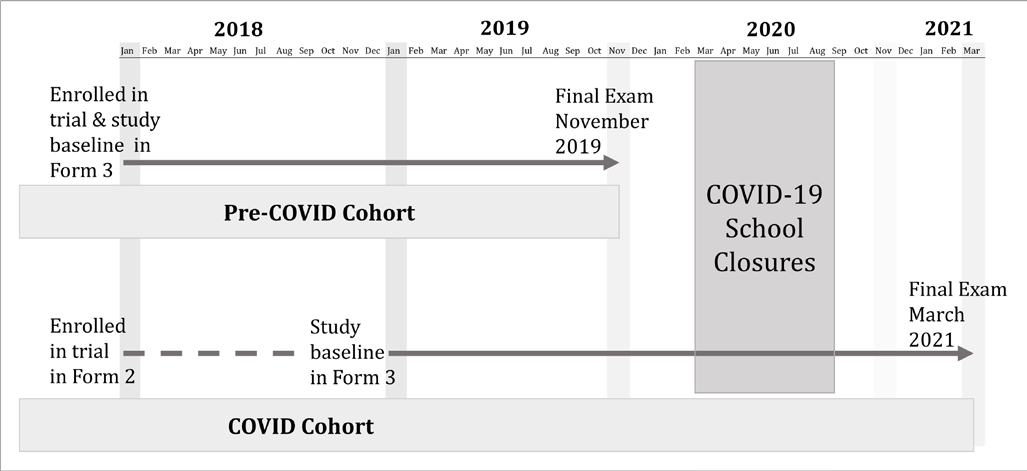
Study participant follow-up diagram. Secondary school in Kenya is comprised of four academic years (Forms 1–4) with Form 3 being students’ penultimate year and Form 4 being the final academic year in which students sit their final examinations.

This study is nested in the CCG Trial which evaluated the effect of conditional cash transfer and/or menstrual cups on a composite of deleterious outcomes, detailed elsewhere.^18^ The trial included 96 secondary schools across Siaya County; the 12 trial schools in Rarieda subcounty selected for this study were enrolled in 2018, uniquely positioning them to follow-up students graduating prior to and after the COVID-19 lockdown. The 910 participants studied here were enrolled between May and July 2018; of these, 403 girls were attending their penultimate/third year of secondary school (‘Form 3’) and sat their final Kenya Certificate of Secondary School (KCSE) examinations in November of 2019 and 507 girls were attending their second year of secondary school (‘Form 2’) and anticipated completing their final academic year (‘Form 4’) in November 2020. Due to the school closures, this second group sat their final examinations 4 months late in March 2021 (figure 1).

In this analysis, we use a causal-comparative design to compare pregnancy and schooling outcomes between girls who experienced school closures (ie, the ‘COVID-19’ cohort) and girls who graduated the year prior (ie, the ‘pre-COVID-19’ cohort). Study questionnaires were administered annually to study participants. For comparability, the survey at the initiation of girls’ penultimate year of secondary school, Form 3, is used for both groups to assess baseline study conditions. While schools did not reopen until January 2021 for the majority of learners, students in their last academic year (Form 4) were allowed back to school in October 2020 to prepare for their final examinations. End of study surveys were conducted at school during participants’ last term of study prior to examinations: between May and July 2019 for the pre-COVID-19 cohort and between October and December 2020 for girls in the COVID-19 cohort. If COVID-19 containment measures prevented study staff from entering a school, a location near to the school, such as a church or community hall, was used for follow-up visits with school attending participants informed via the school and all others informed via phone.

Female day scholars who were resident of the area were attending 1 of the 12 selected schools and in the designated class years at enrolment, had reached menarche, were not visibly or declared pregnant at enrolment, had no disability precluding participation and had informed parent–guardian consent and gave individual assent to participate were eligible for the cRCT.^18^ Enrolled participants were invited to complete a self-administered sociobehavioural questionnaire on an Android tablet which could be completed in English or the local language (Dholuo). Survey data were deidentified at the source. Survey tools were prevalidated and adapted from existing tools, described elsewhere.^19^ COVID-19-related stress was assessed using eight questions from Mental Health Impacts (Module 6) of the Johns Hopkins University COVID-19 Community Response Survey subscale.^20^ Questions that were most relevant to the study setting and population were selected. Response categories were simplified to ‘agree’, ‘disagree’ or ‘don’t know’. Three questions from Module 9 (Violence and Trauma) on violence and crime in participants’ villages and homes were also included.

Girls reporting a prior or current pregnancy and girls who were visibly pregnant or reported pregnant by their schoolteacher were followed up by a study counsellor for documentation of the pregnancy outcome and date of delivery. School dropout and school migration were monitored via school register data, collected termly from all schools to check student attendance and confirmed against teacher report and school visits. For those no longer attending school, home visits were conducted to establish whether girls had discontinued schooling or had transferred to a new school. This follow-up was also done after the completion of the final year examinations to capture if any girls dropped out in their final term. Reason for dropout was also collected during these home visits, including due to pregnancy.

Participation in the trial was entirely voluntary; girls could withdraw from the study at any time.

### Study area and population

The study took place in Rarieda subcounty, Siaya County, western Kenya. The area borders the eastern shores of Lake Victoria and is situated 400 km northwest of the capital Nairobi. The population are mainly subsistence farmers, livestock producers and fisherfolk and are predominantly ethnic Luo.^21 22^ The area is characterised by limited employment opportunities and high levels of outmigration for work and suffers from high endemicity of malaria, HIV, TB and schistosomiasis close to the lakeshores.^21^ Prior to COVID-19, 23.3% of adolescent girls had a history of pregnancy,^23^ and maternal mortality was high with a 1-in-26 lifetime risk of dying from pregnancy-related causes.^19 24^ Two out of every five child learners are estimated to miss school daily in Siaya County.^25^ Gender equity seen in primary school falls during adolescence, with 25%–33% more boys than girls attending secondary school by age 18 years.^13^

### Study measures

The study questionnaire included indicators on household conditions, individual characteristics and sexual activity and behaviours. For COVID-19 group girls who experienced school closures, the follow-up questionnaire contained questions on COVID-19-related changes to household income, perception of violence and crime in the community and individual stress.

Primary response variables included incident pregnancy: girls who became pregnant during Forms 3–4 contributed to this outcome (projected delivery date between 1 October 2018 and 31 August 2020 in the pre-COVID-19 cohort and delivered or had a pregnancy detected after 1 October 2019 in the COVID-19 cohort). For comparability girls who delivered prior to 1 October, dates were coded as having been pregnant prior to the start of the present study and did not contribute to the endpoint. Schooling outcomes were measured with a binary response: (1) school dropout—girls who discontinued their education prior to the completion of their final exam and (2) school migration—girls who transferred schools prior to the completion of their final exam.

Secondary outcomes explored included sexual activity and sexual debut, condom and hormonal contraceptive use, engaging in work for pay and prior-day non-school-related workload measured in hours and sexual violence.

To measure if both groups were comparable at baseline in girls’ third year of secondary school, characteristics were selected due to their established importance in understanding girls’ vulnerability to adolescent pregnancy and school dropout. Covariates explored included individual characteristics (age, alcohol use, sexual activity, condom and contraceptive use and experiences with sexual harassment and violence), family characteristics (marital status, having previously been pregnant, caring for a baby at home, having no living parent), household characteristics (socioeconomic status (SES)) and financial characteristics (work for pay, hours of non-school-related work (ie, hours spent doing laundry, cleaning, farm work or collecting firewood and water) and transactional sex).

Certain characteristics were dichotomised: (1) marital status—‘married, cohabiting, divorced’ versus ‘single, other’; (2) having a baby at home to care for; (3) being harassed for sex in or out of school; (4) having ever been indecently touched; (5) performing work for pay; (6) performing sex for goods or favours and (7) having ever consumed alcohol. Hours of non-school-related tasks or work on the previous day were measured as a continuous variable (ie, number of hours) and restricted to weekdays when girls would also have school. Certain sexual activity measures were dichotomised and measured cumulatively over different survey rounds: (1) self-reported sexual activity, (2) early sexual initiation—before age 15 years, (3) whether first sex was desired and (4) having had a previous pregnancy. Practices measured only at end of study included: (1) incident sexual debut, (2) experiencing intimate partner sexual violence in the past 6 months, (3) condom use in the past 6 months and (4) current use of hormonal contraceptives. Age of sexual debut was maintained as continuous variable (ie, number of years old at debut).

Participant age was calculated as a girl’s age at the start of the Form 3 academic year, dated 2 January. SES and girls’ orphanhood status were measured only once at study enrolment. Orphanhood status was dichotomised into having no living parent—‘orphan’ versus ‘one or both parents alive’. An absolute index for SES was constructed following the methodology of Kabudula *et al*.^26^ SES values were split into five quintiles and dichotomised into households in the lowest two quintiles (quintiles 1–2) and wealthier three (quintiles 3–5).

### Statistical analysis

Descriptive statistics were used to summarise sample characteristics. We assessed comparability of baseline covariates via multilevel mixed effects generalised linear models (GLMMs) with a random effect for school. GLMMs were fitted with a Gaussian distribution and identity link function and assessed at a level of significance of 5% for continuous variables; binary endpoints were modelled using a Poisson distribution with a log link function and a robust SE adjustment. To investigate if COVID-19-related school closures and restrictions were associated with the key binary outcomes of interest, GLMMs were constructed with the same model fitting to estimate risk ratios (RRs), adjusted RRs (aRRs) and their corresponding 95% CIs. We calculated unadjusted RRs by fitting the GLMMs to individual follow-up data with the COVID-19 exposure status as the only study variable. We calculated aRRs by adding participant age, household SES and having a living parent to the unadjusted GLMMs; these factors were selected a priori due to their established association with adolescent pregnancy.^27–29^ Lastly, to compare incidence rates (IRs), we fitted GLMMs with a Poisson distribution, log link function and robust SE, with log follow-up time as an offset. These models were run to evaluate whether or not the magnitude of effect on pregnancy was simply due to longer follow-up in the COVID-19 cohort and to measure the IRs of pregnancy in the COVID-19 cohort prior to and during school closures; schools were closed from 20 March 2020 to 12 October 2020. In this latter group, descriptive statistics were measured to assess reported levels of pandemic-related stress, changing household conditions and community violence in the aftermath of the lockdown. All statistical analyses were performed using STATA MP V.17.0 (StataCorp LP, College Station, Texas, USA).

## Results

**Figure 2:**
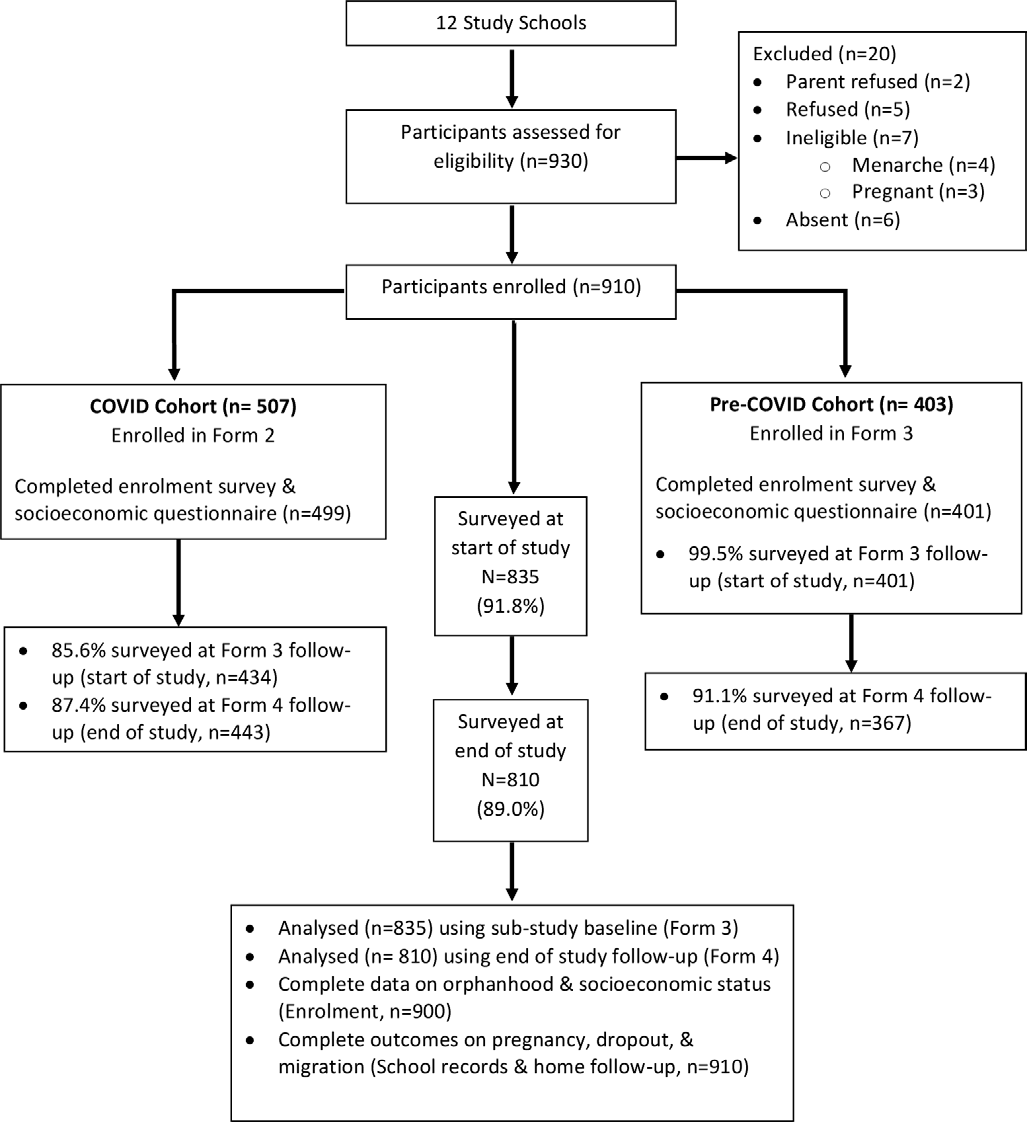
Study participant attrition diagram.

Overall, 910 participants were included in this study, 507 in the COVID-19 cohort and 403 in the pre-COVID-19 cohort. Complete Form 3 survey data were collected for 434 girls in the COVID-19 cohort (85.6%) and 401 in the pre-COVID-19 cohort (99.5%) (figure 2), leading to a comparative Form 3 sample of 835 girls. All 910 girls contributed data on the primary endpoints (pregnancy and dropout) which were collected at the school and home level. Overall, survey retention was 89% at end of study follow-up and was not significantly different between groups (table 1). Differences in self-reported outcome variables between participants lost to survey follow-up (n=100) and those who completed the survey were few: girls who did not sit the end of study survey were more likely to have experienced indecent touching 26.4% versus 15.0% (online supplemental table 1). All analyses of COVID-19-related effects on surveyed outcomes were done on the 810 girls (443 COVID-19 cohort and 367 pre-COVID-19 cohort girls) with end of study follow-up survey data regardless of successful interview in Form 3 (figure 2).

10.1136/bmjgh-2021-007666.supp1 Supplementary data

**Table 1:** Sample characteristics and Form 3 baseline comparability between girls graduating prior to the pandemic and girls experiencing COVID-19-related school closures (n=910)

Baseline characteristics*	COVID-19 cohort (n=509)	Pre-COVID-19 cohort (n=403)	P value
	N (%) or mean (IQR)	N (%) or mean (IQR)	
Age in years (on 2 January of their Form 3 year)	17.5 (16.5–18.4)	17.2 (16.4–17.9)	**<0.001**
Socioeconomic status† (lowest two quintiles)	195 (39.1)	166 (41.4)	0.411
Marital status (MCW)	11 (2.5)	17 (4.2)	0.181
Baby at home to care for	24 (5.5)	20 (5.0)	0.570
Orphan (no living parent)	27 (5.3)	19 (4.7)	0.629
Ever consumed alcohol (self-report)	2 (0.46)	1 (0.25)	0.636
Happy at home	422 (97.2)	390 (97.3)	0.979
Happy at school	425 (97.9)	394 (98.3)	0.654
Work for pay	90 (20.7)	114 (28.4)	0.074
Non-school-related work hours—prior school day‡	2.17 (1.0–3.0)	2.31 (1.0–3.0)	0.341
Harassment for sex—in school	38 (8.8)	34 (8.5)	0.701
Harassment for sex—out of school	122 (28.1)	167 (41.7)	**<0.001**
Being touched indecently—past 6 months	67 (15.4)	64 (16.0)	0.776
Prior pregnancy§	28 (6.4)	21 (5.2)	0.448
Reported sexual activity	165 (38.0)	141 (35.2)	0.378
Age of sexual debut¶	15.8 (15.0–17.0)	15.5 (15.0–17.0)	0.379
Early sexual debut (<15 years)**	20 (4.6)	14 (3.5)	0.268
Wanted to have sex—first time**	54 (32.7)	38 (27.0)	0.282
Reported condom use—past 6 months**	94 (57.0)	91 (64.5)	0.298
Hormonal contraceptives—current use**	16 (9.7)	11 (7.8)	0.550
Engaging in transactional sex	11 (2.5)	7 (1.8)	0.405
Lost to survey follow-up	64 (12.6)	36 (8.9)	0.319

Statistically significant differences at p<0.05 in bold.

*Survey responses at Form 3 were collected for 835 girls (434 COVID-19 and 401 pre-COVID-19).

†10 girls missing data on socioeconomic status, measured as lowest two quintiles versus wealthier three.

‡Only among girls responding to participating in work activities (prior day restricted to Monday-Friday).

§Includes girls whose delivery dates were prior to 1 October of their Form 3 year and girls who reported a prior pregnancy in the survey.

¶Only 150 girls knew their age at sexual debut (63 COVID-19 and 87 pre-COVID-19).

**Only among sexually active.

MCW, married, cohabiting, divorced.

At the start of Form 3, the mean age of participants was 17.2 years (IQR: 16.4–17.9) for girls in the pre-COVID-19 cohort and 17.5 years (IQR: 16.5–18.4) for girls in the COVID-19 cohort, suggesting girls in the COVID-19 cohort were slightly older. Groups were mostly balanced at baseline, with both groups having comparable individual and household characteristics (table 1), with the exceptions of higher rates of reported harassment for sex outside of school (41.7%) and some evidence for higher levels of work for pay (28.4%) in the pre-COVID-19 cohort relative to girls in the COVID-19 cohort.

Among girls experiencing COVID-19 school closures and restrictions, the incidence of dropout was 9.4% versus 3.2% in the pre-COVID-19 cohort (table 2). After adjustment for girls’ age, household SES and orphanhood status, COVID-19 cohort girls had three times the risk of dropout of pre-COVID-19 girls (aRR=3.03; 95% CI: 1.55 to 5.95, p value=0.001). Girls in the COVID-19 cohort also migrated schools at 3.4 times the rate of girls graduating prior to COVID-19 (aRR=3.39; 95% CI: 1.70 to 6.77, p value=0.001). School disruption was coupled with two times the risk of adolescent pregnancy among girls in the COVID-19 cohort versus their pre-COVID-19 peers (aRR=2.11; 95% CI: 1.13 to 3.95, p value=0.019), even after accounting for the person-months of follow-up (IR ratio (IRR): 1.90; 95% CI: 1.02 to 3.52, p value=0.042). Incident pregnancy between the start of Form 3 and completion of examinations was 10.9% among COVID-19 cohort girls versus 5.2% in the pre-COVID-19 cohort. Also, in the COVID-19 cohort, pregnancy incidence (conceptions) was 9 per 100 person-years at risk during the 7 months schools were closed (March to October 2020) (IR: 8.82; 95% CI: 5.91 to 13.16 per 100 person-years at risk) and 5 per 100 person-years at risk in the previous 14 months of observation (January 2019 to March 2020) (IR: 5.19; 95% CI: 3.64 to 7.37 per 100 person-years at risk), indicating heightened pregnancy during these later months (IRR: 1.74; 95% CI: 1.01 to 2.99, p value=0.046).

**Table 2:** Effects of COVID-19 on schooling and sexual activity measures

	COVID-19 cohort	Pre-COVID-19 cohort	RR (95% CI)	P value	aRR (95% CI)	P value
Dropped out of school	49/509 (9.7)	12/403 (3.0)	**3.18 (1.63 to 6.22**)	**0.001**	**3.03 (1.55 to 5.95**)	**0.001**
Migrated schools	36/509 (7.1)	8/403 (2.0)	**3.37 (1.74 to 6.53**)	**<0.001**	**3.39 (1.70 to 6.77**)	**0.001**
Incident pregnancy	55/509 (10.9)	21/403 (5.2)	**2.13 (1.18 to 3.87**)	**0.013**	**2.11 (1.13 to 3.95**)	**0.019**
Sexually active	293/443 (66.1)	186/367 (50.7)	**1.31 (1.11 to 1.54**)	**0.001**	**1.28 (1.09 to 1.51**)	**0.002**
Sexual debut*	135/285 (47.4)	62/243 (25.5)	**1.87 (1.27 to 2.75**)	**0.001**	**1.84 (1.25 to 2.70**)	**0.002**
First sex desired†	52/293 (17.8)	67/186 (36.0)	**0.49 (0.38 to 0.63**)	**<0.001**	**0.49 (0.37 to 0.65**)	**<0.001**
Condom use—last 6 months†	140/293 (47.8)	97/186 (52.2)	0.92 (0.71 to 1.18)	0.504	0.91 (0.70 to 1.18)	0.477
Hormonal contraceptives—current†	12/293 (4.1)	13/186 (7.0)	0.59 (0.24 to 1.47)	0.254	0.58 (0.22 to 1.48)	0.252
Work for pay	105/443 (23.7)	93/367 (25.3)	0.94 (0.74 to 1.19)	0.597	0.93 (0.72 to 1.21)	0.596
Non-school-related work hours—prior school day‡	3.32 (2.0–5.0)	2.63 (1.0–3.0)	**1.85 (1.18 to 2.90**)	**0.007**	**1.92 (1.23 to 2.99**)	**0.004**
Sexual violence	14/443 (3.2)	5/367 (1.4)	2.36 (0.69 to 7.99)	0.169	2.15 (0.65 to 7.16)	0.211
Indecent touching	50/443 (11.3)	54/367 (14.7)	0.77 (0.49 to 1.20)	0.241	0.75 (0.47 to 1.19)	0.225

Statistically significant differences at p<0.05 in bold.

*Only among girls who were not sexually active at baseline.

†Only among sexually active.

‡Only among girls responding to participating in work activities (prior day restricted to Monday-Friday).

aRR, adjusted RR; RR, risk ratio.

Self-reported sexual activity by Form 4 follow-up was reported by 66.1% of the COVID-19 cohort versus 50.7% of their pre-COVID-19 peers (aRR=1.28; 95% CI: 1.09 to 1.51, p value=0.002). Incident sexual debut among girls not sexually active at the start of Form 3 was reported by 47.4% of girls in the COVID-19 cohort versus 25.5% of girls in the pre-COVID-19 cohort (aRR=1.84; 95% CI: 1.25 to 2.70, p value 0.002). Among girls who were sexually active, 36.0% of girls in the pre-COVID-19 cohort reported their first sex as having been desired, while only 17.8% of girls in the COVID-19 cohort reported the same (aRR=0.49; 95% CI: 0.37 to 0.65, p value<0.001). No differences were seen between groups for girls reporting condom use in the past 6 months or current use of hormonal contraceptives (table 2).

Girls in the COVID-19 cohort reported similar rates of performing work for pay as their pre-COVID-19 cohort peers; however, the number of hours worked in the prior school day was significantly higher (3.32 hours vs 2.63 hours), indicating a heightened non-school-related workload at home (aRR=1.92; 95% CI: 1.23 to 2.99, p value=0.004). Participants’ reported exposure to indecent touching and sexual violence was comparable between the two groups.

### Pandemic-related measures and stress among girls experiencing COVID-19 lockdowns

**Figure 3:**
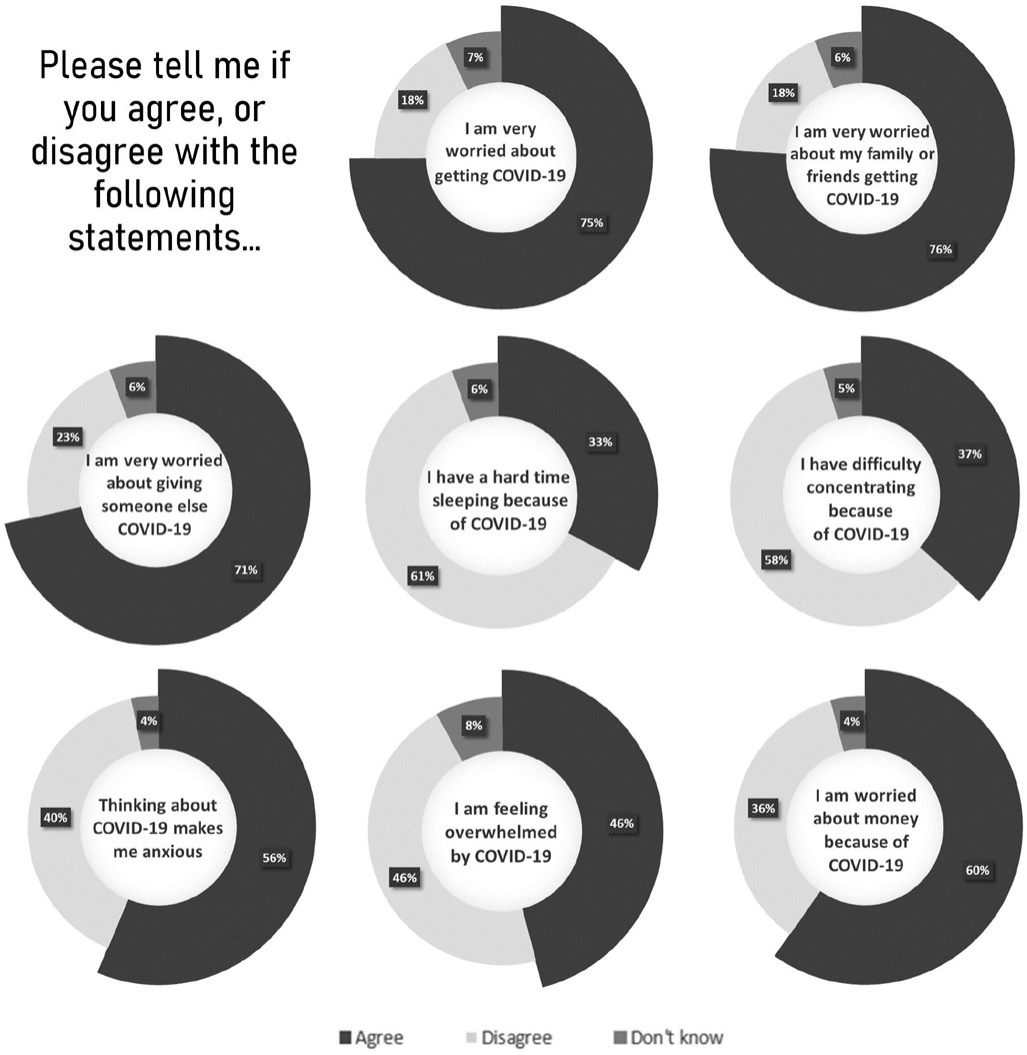
Reported mental health impacts due to COVID-19 (n=442). Per cent of girls reporting stress indicator.

At end of study, among the 443 girls who experienced the COVID-19 containment measures, 442 completed a COVID-19-related questionnaire which captured information on pandemic-related changes in economic conditions, violence and individual stress. Of these girls, 80.5% reported their household income decreased during the pandemic, with 41.7% of previously employed girls reporting losing their source of income. The majority of girls who experienced COVID-19-related restrictions voiced worrying about money (59.7%) and running out of food (68.1%). Girls reported elevated stress due to COVID-19, with most girls worried about contracting or transmitting the disease (figure 3).

A sizeable percentage of girls reported an increase in violence (36.2%) and crime (43.4%) in their communities after COVID-19; 36.2% of girls in the COVID-19 cohort reported feeling less safe at home.

## Discussion

The COVID-19 school closures and containment measures notably impacted the daily life of vulnerable adolescent schoolgirls in western Kenya. This study follows girls from the start of their third year of secondary school through to the conclusion of their final school examinations to measure COVID-19’s impact on their sexual health and schooling. Girls, whose schooling was disrupted due to COVID-19, experienced a threefold risk of dropping out of school and 3.4 times the risk of changing schools relative to their peers. Girls who experienced school closures also had twice the risk of falling pregnant prior to completing school, with 1 in 10 COVID-19 cohort girls becoming pregnant prior to sitting their examinations. This group also saw increased sexual activity, with nearly one in two COVID-19 cohort girls becoming sexually active during follow-up. These girls were twice as likely to report their first sex was not desired when compared with girls who did not experience COVID-19 during secondary school. Girls in the COVID-19 cohort also reported increased work hours and four out of every five girls in this group reported their household income decreased during the pandemic.

Our results show that adolescent girls in this study area, which typifies many rural areas of sub-Saharan Africa, are particularly vulnerable to sexual and reproductive harms during emergencies. These findings are in line with other studies that have shown heightened rates of adolescent pregnancies after population-level crises. For example, during the west-African Ebola outbreak, in Sierra Leone teenage pregnancies increased by 25% and were hypothesised to represent underlying vulnerabilities to transactional sex and sexual exploitation.^15^ While reported transactional sex was too low to detect in our study, our findings showed that girls experiencing COVID-19 restrictions were twice as likely to report that their first sex was undesired, indicating a possible rise in sexual coercion. Studies have hypothesised that, during emergencies, increased economic strain may push girls to engage in sex in exchange for money or favours.^30^ Wider studies have also shown that girls’ facing sexual coercion or abuse obtain lower schooling and lifetime earnings,^31^ with adolescent pregnancy itself being a major driver of school dropout.^11^ These outcomes underscore the longevity of effect resulting from harms experienced during adolescence.

Our study highlighted the detrimental and potentially long-term effects COVID-19 containment measures had on girls schooling. Girls enrolled in secondary school during COVID-19 were 3.4 times more likely to change schools relative to their non-COVID-19 peers. In addition to being disruptive to education, among adolescents, domestic migration has been associated with unstable living arrangements and early engagement in high-risk sexual activity and informal work.^32^ Girls in the COVID-19 cohort were also three times as likely to drop out of school. During Ebola many countries employed school closures as a policy response to contain the outbreak; however, long after schools reopened, school enrolment of young girls remained significantly lower than pre-Ebola.^33 34^ These impacts disproportionately affect lower income and more marginalised students and particularly older adolescents who take on additional paid or unpaid work during crises.^35 36^ Studies have shown that once engaged in economic activities, older adolescents may have to retain these responsibilities even after schools reopen to help sustain their families.^30 35 36^ Our results echo these findings, showing how girls engaged in non-school-related work had increased work hours in the aftermath of the COVID-19 pandemic.

Schools act as a social vaccine in preventing adolescent SRH harms, including pregnancy.^11–13^ In late secondary school, many students transition into full-time boarders in Kenya; thus, the closure of schools may have affected girls’ living environment in addition to their learning environment. This was not unique to Kenya: school closures affected an estimated 80% of young learners globally, most acutely affecting students in low-income countries that had the fewest resources to support remote learning. However, in higher-income countries, where much of schooling was moved online and learning was expected to continue, inequalities were also found to widen, leaving behind students with limited access to internet, no suitable place to do homework and those from households with unstable housing. In the Netherlands, for example, one study found that students from disadvantaged households experienced 60% more learning loss than those in the general population;^37^ another study in Canada is projecting that the student skills gap due to socioeconomic factors in adolescents will widen by more than 30%.^38^ In the USA, where school closures were not uniform across states, one study found that school closures were higher among lower-income schools with higher numbers of minority students and higher numbers of students who were eligible for free lunches.^38^ Evidence is growing that food instability among youth is rising due to school closures. In India, for example, where over half of all children are undernourished, the Mid-day Meal, a school-based nutrition programme provided minimum calorie and protein requirements to 80% of primary school students nationwide prior to school closures.^39^ Moreover, schools serve as the primary link between adolescents and child protective services. Concern is rising that school closures are leaving adolescents to suffer without support at a time when mental health outcomes have deteriorated in adolescents.^40^ A recent study in the USA identified that child maltreatment reports dropped during COVID-19, possibly highlighting a break in the referral chains for vulnerable children and youth.^41^ Our findings add to this mounting body of evidence, which warns of rising inequalities due to COVID-19-related school closures, and emphasise the multifaceted role schools play in adolescent health.

In Kenya, increased domestic abuse within households has been reported, partly due to pandemic-related stress and income loss.^42^ In our study, girls voiced decreasing household incomes, feeling less safe at home and increasing crime in their communities in the aftermath of the pandemic. Girls also reported heightened pandemic-related stress. This is in line with what has been documented worldwide: studies have evidenced higher levels of anxiety among females, younger individuals, those of lower incomes and those who experienced loss of income.^43–46^

Some limitations of this study should be considered. First, girls in the COVID-19 cohort were followed up for an additional four months due to their delayed KCSE examination. This unequal follow-up may have given them additional time to fall pregnant relative to their pre-COVID-19 peers. In response we employed person–time analysis to adjust for this unequal follow-up. To note, because this study was completed immediately after the COVID-19 cohort girls sat their final examinations, girls who were in the early months of their pregnancy and sat their examination may have been missed. Consequently, the estimated pregnancy prevalence in the COVID-19 cohort may be an underestimate of all girls who became pregnant while still enrolled in secondary school. Because the trial was still active in the study area for a year after pre-COVID-19 girls completed their school, all pregnancy outcomes occurring during school were documented in full. Second, behavioural data were self-reported and certain sensitive indicators, such as sexual activity and sexual behaviours, may be under-reported. We also resensitised girls at every survey round on the importance of truthful reporting which may have improved response bias but led to differential reporting between survey rounds. In response, we took a cumulative measure of girls reporting sexual activity at any survey. Third, this study only measured effects in a secondary school-going population and may not reflect vulnerability to harms of other populations such as out-of-school girls. Lastly, this study was embedded within a cRCT that was testing different interventions to improve girls schooling and SRH harms, with girls in the COVID-19 cohort experiencing interventions for an additional year relative to their pre-COVID-19 comparators. We note that any beneficial impact from being in the trial longer would push the measure of effect in the COVID-19 cohort towards the null, suggesting the results presented here may be conservative estimates of the pandemic’s effects on girls’ health and schooling relative to non-trial schoolgirl populations.

## Conclusion

Our study provides important insights into the SRH and schooling consequences of the COVID-19 pandemic on a vulnerable population of originally school-going adolescent girls in rural Kenya. We found that the COVID-19 pandemic and related school closures had significant harmful effects on schooling and girls’ sexual activity, including increased pregnancy incidence and possible sexual coercion. These harms, occurring during adolescence, could have lasting consequences well after the pandemic subsides. Country reliance on school closures as a primary mechanism to abate infectious disease outbreaks must take into consideration the resulting side effects that occur. Appropriate programmes and interventions that help buffer the effects of population-level emergencies on vulnerable adolescents are warranted.

## Data Availability

This study was conducted with approval from the Kenya Medical Research Institute (KEMRI) Scientific and Ethics Review Unit (SERU) which requires that data should be released from any KEMRI-based Kenyan study (including deidentified data) only after written approval for additional analyses. In accordance, data for this study will be available upon request, after obtaining written approval for the proposed analysis from the KEMRI SERU. Their application forms and guidelines can be accessed at https://www.kemri.org/seru-overview. To request these data, please contact the KEMRI SERU at seru@kemri.org.
