# Supplementary material for: Impact of COVID-19 lockdowns on adolescent pregnancy and school dropout among secondary schoolgirls in Kenya
Source: BMJ Glob Health. 2022 Jan 13;7(1):e007666. doi: 10.1136/bmjgh-2021-007666 (PMC8761596; doi:10.1136/bmjgh-2021-007666)
Supplement: Supplementary data [file bmjgh-2021-007666supp001.pdf]

S. Table 1: Sample characteristics of girls who missed the end of study survey (n=100)

**Baseline characteristics†**	**Lost to follow-up N (%) or mean (IQR)**	**Completed follow-up N (%) or mean (IQR)**	**p-value**
Age in years (on Jan 2 of their Form 3 year)	17.6 (17.4-17.9)	17.4 (17.3-17.5)	0.272
Socioeconomic status* (lowest two quintiles)	43 (43.0)	318 (39.3)	0.567
Marital status (MCW/SO)	2 (3.8)	26 (3.3)	0.868
Baby at home to care for	5 (9.4)	39 (5.0)	0.193
Orphan (no living parent)	4 (4.0)	42 (5.2)	0.535
Work for pay	43 (81.1)	588 (75.2)	0.248
Non-school-related work hours – prior school day‡	2.61 (1.0-3.0)	2.22 (1.0-3.0)	0.205
Being touched indecently – past 6 months	14 (26.4)	117 (15.0)	**0.008**
Reported sexual activity	24 (45.3)	282 (36.1)	0.140
Wanted to have sex – first timeº	7 (29.2)	85 (30.1)	0.890
Reported condom use - past 6 months º	15 (62.5)	170 (60.3)	0.783
Hormonal contraceptives - current use º	3 (12.5)	24 (8.5)	0.562
Engaging in transactional sex	2 (3.8)	16 (2.1)	0.425

Footnote: Abbreviations: MCW – married, cohabiting, divorced; statistically significant differences at p<0.05 in **bold;** †47 girls missed both survey rounds; *10 girls missing data on socioeconomic status, measured as lowest two quintiles vs wealthier three; ‡Only among girls responding to participating in work activities (prior day restricted to M-F); ºonly among sexually active; ^includes girls whose delivery dates were prior to Oct 1 of their Form 3 year and girls who reported a prior pregnancy in the survey
